# Identification of a capsular variant and characterization of capsular acetylation in *Klebsiella pneumoniae* PLA-associated type K57

**DOI:** 10.1038/srep31946

**Published:** 2016-08-23

**Authors:** Chun-Ru Hsu, Chun-Hsing Liao, Tzu-Lung Lin, Han-Ru Yang, Feng-Ling Yang, Pei-Fang Hsieh, Shih-Hsiung Wu, Jin-Town Wang

**Affiliations:** 1 Department of Microbiology, National Taiwan University College of Medicine, Taipei, Taiwan; 2 Department of Medical Research, E-Da Hospital, Kaohsiung, Taiwan; 3 School of Medicine, I-Shou University, Kaohsiung, Taiwan; 4 Department of Internal Medicine, Far Eastern Memorial Hospital, New Taipei City, Taiwan; 5 Institute of Biological Chemistry, Academia Sinica, Taipei, Taiwan; 6 Department of Internal Medicine, National Taiwan University Hospital, Taipei, Taiwan

## Abstract

*Klebsiella pneumoniae* can cause community-acquired pyogenic liver abscess (PLA). Capsular polysaccharide (CPS) is important for its virulence. Among 79 capsular (K) types discovered thus far, K57 is often associated with PLA. Here, we report the identification of a K57 variant. *Cps* gene locus sequencing revealed differences between the K57 reference strain 4425/51 (Ref-K57) and a variant, the PLA isolate A1142. While Ref-K57 *cps* contained *orf13* encoding a putative acetyltransferase, the insertion of a putative transposase-encoding gene at this position was detected in A1142. This variation was detected in other K57 clinical strains. Biochemical analyses indicated that A1142 was deficient in CPS acetylation. Genetic replacement and complementation verified that *orf13* was responsible for CPS acetylation. Acetylation increased CPS immunoreactivity to antiserum and enhanced *K. pneumoniae* induction of pro-inflammatory cytokines through JNK and MAPK signaling. While acetylation diminished the serum resistance of bacteria, it promoted adhesion to intestinal epithelial cells possibly via increasing production of type I fimbriae. In conclusion, acetylation-mediated capsular variation in K57 was observed. Capsular acetylation contributed to the variety and antigenic diversity of CPS, influenced its biological activities, and was involved in *K. pneumoniae*-host interactions. These findings have implications for vaccine design and pathogenicity of *K. pneumoniae*.

*Klebsiella pneumoniae* is an important human pathogen in hospital-acquired and community-acquired infections^1^,^2^,^3^,^4^. This organism causes nosocomial infections, such as septicemia, pneumonia, urinary tract infections (UTIs), surgical site infections and catheter-related infections. *K. pneumoniae* also causes community-acquired infections, such as pyogenic liver abscess (PLA) complicated by meningitis and endophthalmitis, UTIs, and pneumonia. Over the last 20 years, community-acquired *K. pneumoniae* PLA has become an emerging infectious disease worldwide, especially in East Asian countries^5^,^6^,^7^,^8^. This new type of invasive disease is often complicated by metastatic infections, such as meningitis and endophthalmitis. Furthermore, diabetes mellitus, a predisposing factor, has been detected in about 50% of patients with PLA^4^,^9^,^10^.

One of important virulence factors of *K. pneumoniae* is the capsule, an extracellular polysaccharide structure that protects bacteria from lethal serum factors and phagocytosis. At least 79 capsular types have been defined in *Klebsiella*, each representing a distinct chemical structure of the capsular polysaccharide (CPS; the K antigen). The capsular types have been related to *K. pneumoniae* infection and disease severity^11^,^12^. *K. pneumoniae* strains with the K1 and K2 capsular types are identified as the predominant virulent types and also are strongly associated with strains causing PLA^8^,^13^,^14^,^15^. In addition to K1 and K2, other K types are also implicated in PLA. Our previous studies of 42 *K. pneumoniae* strains causing PLA identified those with K1 (n = 35), K2 (n = 2), K57 (n = 2), K5 (n = 1), and K54 (n = 1) capsules, as well as a new type (n = 1)^14^. Similarly, the prevalence of 50 liver abscess isolates in Southern Taiwan revealed capsular types K1, K2, K5, K20, K54, and K57, in addition to an unidentified type^16^. The chromosomal *capsular polysaccharide synthesis (cps*) gene cluster encodes the majority of the proteins involved in the translocation and assembly of surface polysaccharides, composed of repeated sugar subunits^17^, for the *K. pneumoniae* capsule. Genotyping of *cps* can be used to distinguish capsular types^18^,^19^. Information about disease-related capsular types of bacterial pathogens can contribute to diagnosis and to the development of capsule-based vaccines.

To understand pathogen-host interactions and host responses, characterization of the structures and biological activities of various capsular architectures is important. Polysaccharide modifications have been described to cause capsular variation in what were originally defined as singular capsular types in some pathogens, such as *Streptococcus pneumoniae*^20^ and *Escherichia coli* K1 strains^21^. Capsular modifications also may be associated with the virulence of some bacterial strains^21^,^22^. Although modifications of CPS by O-acetyl and O-pyruvyl groups have been reported in a *K. pneumoniae* K1 PLA strain^23^, analysis of potential capsular variation and related modifications in *Klebsiella* is incomplete. In addition, the roles of capsular modifications in *K. pneumoniae* remain to be elucidated. In addition, direct links between the structural, biochemical, and genetic data for some capsular types are still lacking.

*K. pneumoniae* K57 is one of the PLA-associated capsular types. In this study, we discovered the presence of a capsular variant in the K57 capsular type, which was based on genetic data of the *cps* region and biochemical analysis of CPS modification. Our group previously published the complete sequence of the K57 *cps* cluster of the PLA isolate, A1142^14^. Sequencing of the *cps* cluster of another strain, the K57 reference strain (Ref-K57), revealed differences between the two strains at the site of a *cps* gene (*orf13*) encoding a putative acetyltransferase. Variations of this gene were also detected in other K57 clinical strains. By gene replacement and complementation analyses, we verified that *orf13* is responsible for CPS acetylation, which altered *K. pneumoniae* K57 antigenicity, innate host response, serum resistance, and cell adhesion.

## Results

### Identification of differences in *cps* gene loci in *K. pneumoniae* K57 strains

**Figure 1:**
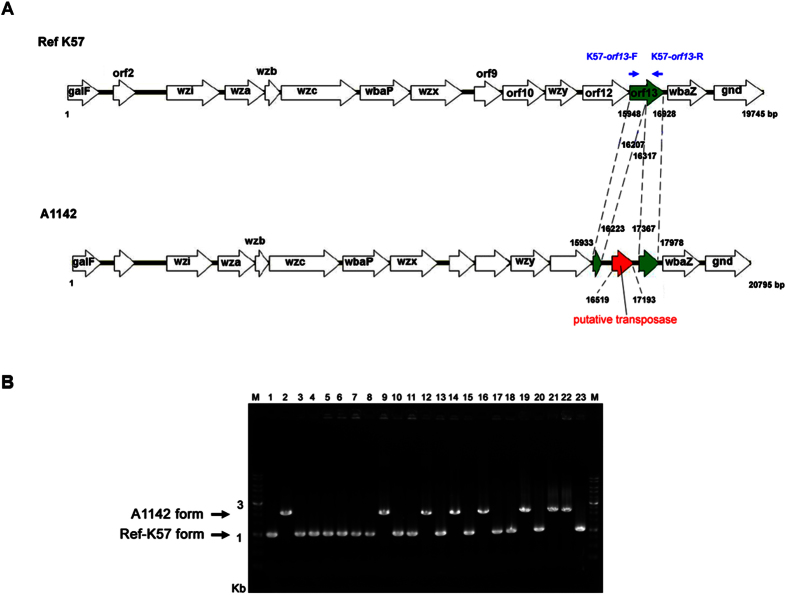
Variation of *orf13* in K57 capsular polysaccharide synthesis (*cps*) regions. (**A**) Comparison of the *cps* between the K57 reference strain (Ref-K57) and A1142. Open reading frames (ORFs) are shown as arrows. Ref-K57 (upper) contains a 981-bp *orf13* (green, residue 15948–16928) encoding a putative acyltransferase family protein. In the PLA isolate A1142 (lower), this gene is interrupted by a gene encoding a putative transposase (red, residue 16519–17193). The blue arrows indicate the positions of the *orf13* PCR primers (K57-*orf13*-F and K57-*orf13*-R) used for panel B analysis (see below). (**B**) PCR analysis of *orf13* in a total of 23 *K. pneumoniae* K57 strains. No. 1: strain Ref-K57; No. 2: strain A1142; No. 3–23: other collected K57 clinical strains. The arrows indicate two forms of *orf13*. M: DNA marker.

Our previous study focused on the *cps* regions of *K. pneumoniae* K57 strain, which is related to PLA^14^. Thus, we sequenced and analyzed the *cps* gene cluster of the K57 reference strain (Ref-K57) from the Statens Serum Institute. We compared the sequence of the Ref-K57 *cps* with that published for the PLA isolate, A1142, another K57 strain (Table 1). We noted an obvious difference in the region between *orf12* and *wbaZ* (Fig. 1A). Specifically, the Ref-K57 sequence included a 981-bp *orf13* (DNA residues 15948–16928) in this position; the predicted *orf13* gene product exhibited 38% amino acid identity (75/196) with the acyltransferase superfamily of proteins (WP_014751172). In contrast, the corresponding gene in A1142 apparently was disrupted by the insertion of a gene encoding a putative transposase; the nominal *orf13* of A1142, thus, was split into two fragments (residues 15933–16223 and 17367–17978). This difference revealed that Ref-K57 and A1142 harbored distinct *orf13* in the *cps* gene loci.

We next examined other *K. pneumoniae* K57 clinical isolates for the presence of similar *orf13* variations. PCR analysis of a total of 23 distinct K57 strains revealed that *orf13* was present in either of two conformations in these strains (Fig. 1B). Specifically, 15 strains (65.2%) harbored *orf13* sequences of a length close to that of Ref-K57 (981 bp). The other eight strains (34.8%) carried *orf13* sequences of a length similar to that of A1142 (2,046 bp). These results indicated that *cps* variation in gene *orf13* was present in a range of different K57 clinical strains.

### Ref-K57 *orf13* is responsible for CPS acetylation, and A1142 generates a K57 variant deficient in CPS acetylation

**Figure 2:**
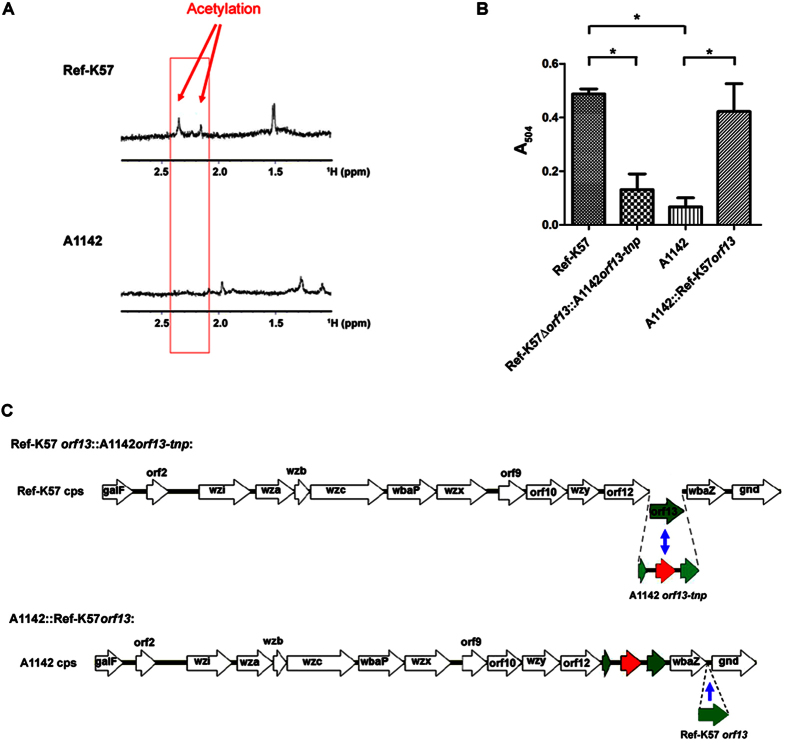
Comparison of CPS acetylation in K57 strains. (**A**) ^1^H NMR spectra of extracted CPS in Ref-K57 (upper) and A1142 (lower). To avoid the effects of sugars from lipopolysaccharide (LPS), O-antigen-lacking Δ*wecA* mutants were analyzed (see methods). The arrows indicate acetylation in Ref-K57. (**B**) Quantification of CPS acetylation. Acetylation levels of extracted CPS from Ref-K57, Ref-K57::*orf13*::A1142*orf13-tnp*, A1142, and A1142::Ref-K57*orf13* were determined by measuring the absorbance at 504 nm and normalized to 50 μg of CPS for each sample. Data are mean ± SEM from three independent experiments. *P < 0.05, Student’s t test. (**C**) The upper diagram shows the genetic replacement of *orf13* in the Ref-K57 *cps* locus (strain Ref-K57Δ*orf13*::A1142*orf13-tnp*). Ref-K57 *orf13* was replaced by the corresponding gene from A1142, in which *orf13* was interrupted by a gene encoding a putative transposase (A1142*orf13-tnp*). The lower diagram shows genetic complementation of *orf13* in the A1142 *cps* locus (strain A1142::Ref-K57*orf13*). For this construct, the *orf13* of Ref-K57 was inserted into the intergenic region between *wbaZ* and *gnd* of the A1142 *cps* locus.

The potential acetylation of K57 CPS isolated from Ref-K57 and A1142 was initially determined and compared. ^1^H NMR analysis showed that CPS extracted from Ref-K57 harbored an acetyl group in the region near 2.0 ^1^H ppm (Fig. 2A, upper panel); this modification was not detected in CPS isolated from A1142 (Fig. 2A, lower panel). Using the Hestrin method to determine the degree of CPS acetylation^24^,^25^, Ref-K57 CPS acetylation levels were higher than those observed for A1142 CPS (Fig. 2B). The main sugar compositions of the CPS polymers from Ref-K57 and A1142 were also compared; no obvious differences were found. According to the published structure of K57 CPS^26^, the ratio of mannose (Man) and galactose (Gal) was predicted to be 2:1. The Man:Gal ratio in both Ref-K57 CPS and A1142 CPS were nearly 2:1. Thus, the CPS polymers of Ref-K57 and A1142 differed in their acetyl modification rather than in their major sugar composition.

**Figure 3:**
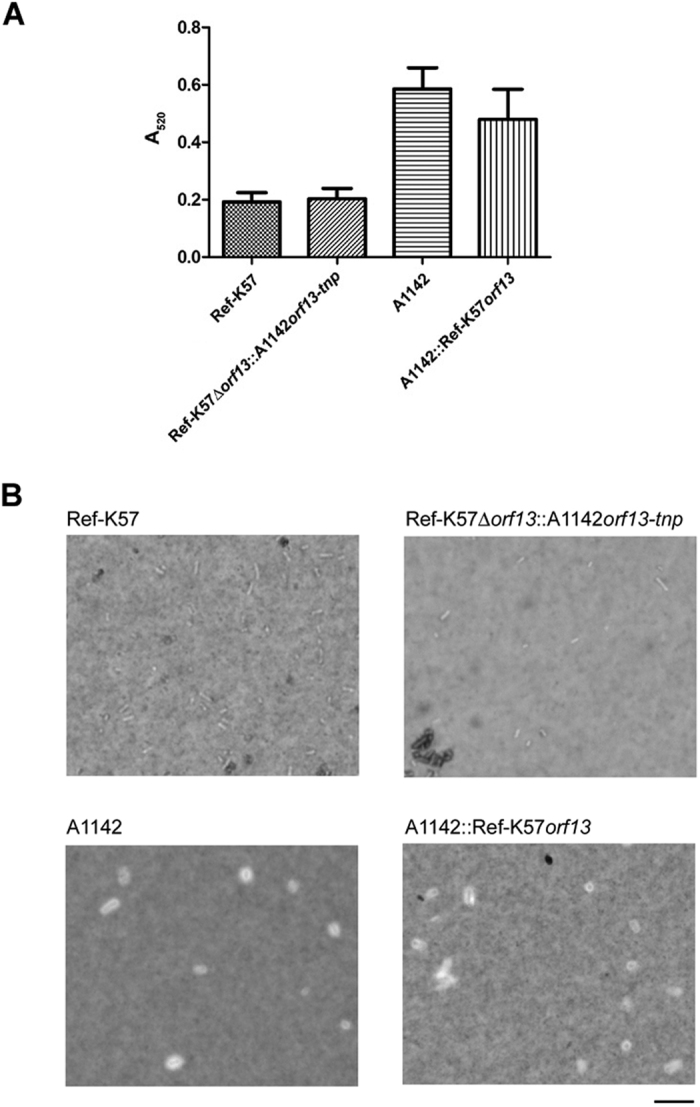
CPS production and capsule staining of *K. pneumoniae* K57 strains. (**A**) Quantification of K57 CPS production. The levels of extracted CPS from the equivalent amounts of overnight cultures of Ref-K57, Ref-K57Δ*orf13*::A1142*orf13-tnp*, A1142, and A1142::Ref-K57*orf13* strains were detected by measuring the absorbance at 520 nm. Data are presented as mean ± SEM from three independent experiments. Ref-K57 vs. Ref-K57Δ*orf13*::A1142*orf13-tnp, P* = 0.8; A1142 vs. A1142::Ref-K57*orf13, P* = 0.45; Ref-K57 vs. A1142, *P* = 0.0079 (Student’s *t* test). (**B**) Capsule staining of *K. pneumoniae*. A drop of log-phase *K. pneumoniae* (as indicated) was stained with India ink and observed using a light microscope. Scale bar is 100 nm.

To verify the function of the *orf13* gene, we constructed gene replacement and complementation K57 strains (Fig. 2C). Specifically, we replaced the *orf13* coding region (residues 15948–16928) of Ref-K57 with the transposase-interrupted *orf13* from A1142 (residues 15933–17975), yielding an isogenic mutant strain that we designated Ref-K57Δ*orf13*::A1142*orf13-tnp* (the “replacement” strain). We generated chromosomal complementation of *orf13* by insertion of the intact Ref-K57 *orf13* gene into the intergenic region between *wbaZ* and *gnd* in the A1142 *cps* cluster, yielding a strain that we designated A1142::Ref-K57*orf13* (the “complementation” strain). This gene replacement or complementation did not cause significant changes in CPS production compared to that in the respective parent strain, as determined by quantification of uronic acid in K57 CPS (Fig. 3A). Ref-K57 generated less CPS than A1142. The replacement strain Ref-K57Δ*orf13*::A1142*orf13-tnp* produced an amount of CPS close to that of Ref-K57, and similarly, no significant differences of CPS production were found between A1142 and A1142::Ref-K57*orf13*. In addition, India ink capsule staining showed no obvious differences in capsule thickness between the gene replacement or complementation strains and the respective parent strains (Fig. 3B). By microscopic observation, Ref-K57Δ*orf13*::A1142*orf13-tnp* looked similar to Ref-K57, and both the A1142 parent and A1142::Ref-K57*orf13* strains had thick capsules surrounding the bacterial cells.

Analysis of CPS acetylation levels (Fig. 2B) revealed that compared to Ref-K57, CPS acetylation of Ref-K57Δ*orf13*::A1142*orf13-tnp* (the replacement strain) was decreased. On the other hand, A1142::Ref-K57*orf13* (the complementation strain) exhibited increased CPS acetylation compared to the A1142 parent. These results indicated that *orf13* in K57 was responsible for CPS acetylation, and was inactivated by disruption of the gene in A1142.

### Acetylation enhances the immunoreactivity of K57 CPS

**Figure 4:**
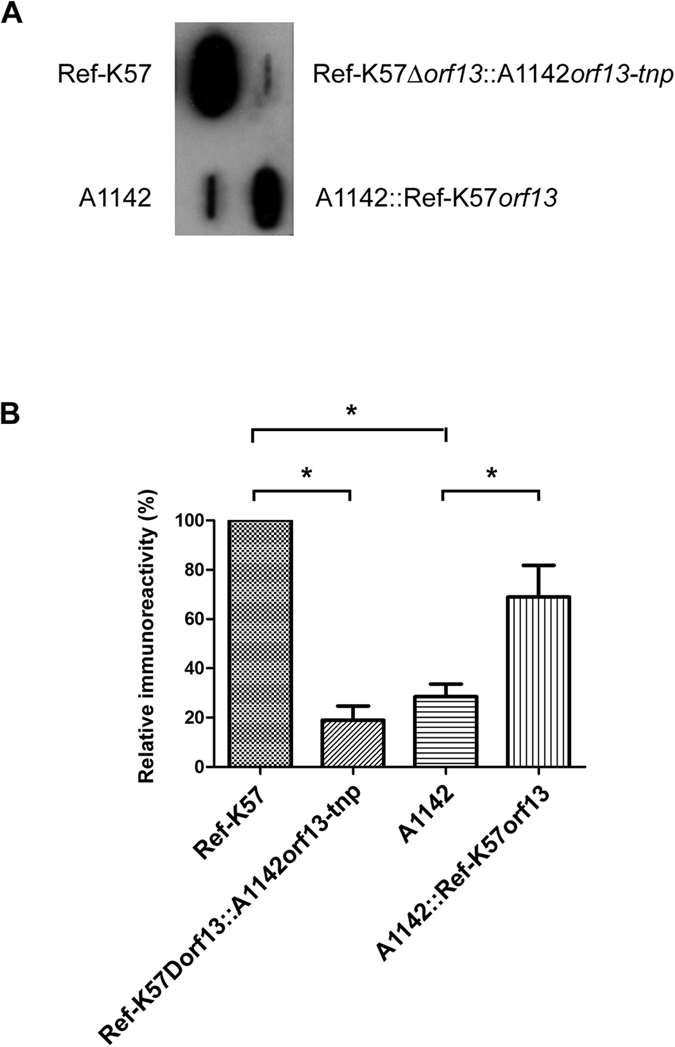
Effects of acetylation on the immunoreactivity of K57 CPS. (**A**) Representative immunoblot of *K. pneumoniae* CPS against anti-K57 antiserum. Equivalent amounts of extracted CPS (2.5 μg per sample) from K57 strains, including Ref-K57, the replacement strain (Ref-K57Δorf13::A1142*orf13-tnp*), A1142 and the acetylation complementation strain of A1142 (A1142::Ref-K57*orf13*), was analyzed against commercial rabbit anti-Ref-K57 antiserum from the Statens Serum Institute. (**B**) Quantification of the immunoreactivity of different K57 CPS (as indicated). Bands on the same immunoblot were densitometrically analyzed using Image J software. Relative activity was presented by comparing to Ref-K57 (set as 100%). Data are mean ± SEM from three independent experiments. *P < 0.05, Student’s t test.

The surface carbohydrate comprising the *K. pneumoniae* capsule typically exhibits strong antigenicity. We, therefore, investigated whether acetylation affected the immunoreactivity of K57 CPS. Immunoblot analysis of equivalent amounts of CPS from each strain was undertaken using commercial anti-K57 serum, which was generated using the Ref-K57 strain by the Statens Serum Institute. As shown in Fig. 4A, both Ref-K57 and A1142 CPS reacted to the anti-Ref-K57 antiserum; however, the Ref-K57 CPS had stronger reactivity. Compared to Ref-K57 CPS, Ref-K57Δ*orf13*::A1142*orf13-tnp* CPS exhibited decreased immunoreactivity, suggesting that acetylation was important for Ref-K57 CPS recognition by anti-Ref-K57 antiserum. Interestingly, A1142::Ref-K57*orf13* CPS exhibited stronger reactivity than parental CPS, indicating that acetylation complementation enhanced the reactivity to anti-Ref-K57 antiserum. To quantify the immunoreactivity, signals derived from the same immunoblot were analyzed by densitometry (Fig. 4B). In Ref-K57Δ*orf13*::A1142*orf13-tnp*, gene replacement reduced the immunoreactivity of Ref-K57 CPS (set as 100%) to ~19%. Compared to Ref-K57 CPS, A1142 CPS exhibited lower immunoreactivity (~29%), which was increased to ~69% in A1142*orf13-tnp*.

### CPS acetylation enhances induction of pro-inflammatory cytokines by K57 *K. pneumoniae*

**Figure 5:**
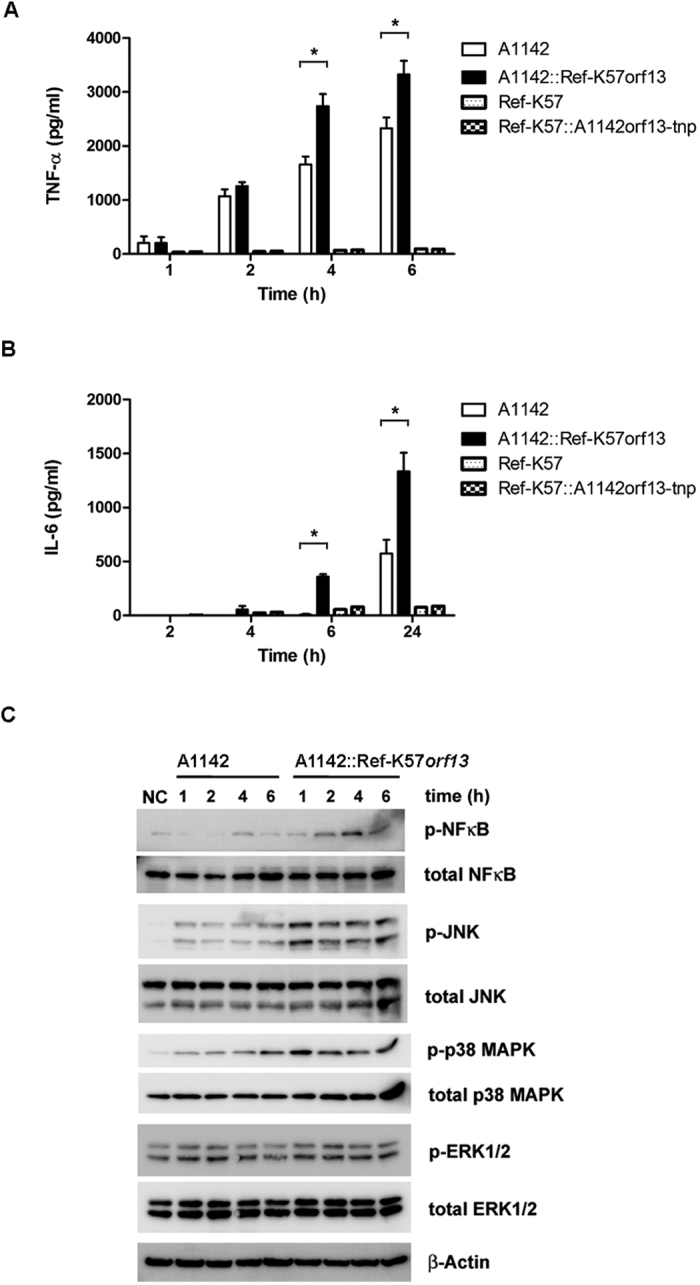
Effects of acetylation on cytokine induction by K57 *K. pneumoniae*. Induction of pro-inflammatory cytokines: (**A**) TNF-α (**B**) IL-6. Mouse macrophage RAW 264.7 cells were incubated with A1142, A1142::Ref-K57*orf13*, Ref-K57, or Ref-K57Δ*orf13*::A1142*orf13-tnp*. The levels of secreted cytokines in the medium were measured at the indicated time points. Data are presented as mean ± SEM from three independent experiments. *P < 0.05, Student’s t test. (**C**) Western blotting of cell signaling induced by A1142 or A1142::Ref-K57*orf13*. Total protein lysates of Raw246.7 cells were subjected to detection for phosphorylation status of NF-κB, JNK, p38 MAPK, and ERK1/2.

Microbial infection often triggers the production of host pro-inflammatory cytokines. The potential impact of K57 *K. pneumoniae* on host innate responses was assessed. The pro-inflammatory cytokines, tumor necrosis factor-α (TNF-α) and interleukin-6 (IL-6), are secreted primarily by activated macrophages, mediating multiple biological effects, including activation of immune responses. A1142 stimulated TNF-α and IL-6 secretion by mouse macrophage Raw 264.7 cells as detected at ~1 and ~6 h after infection (Fig. 5A,B). Ref-K57 did not efficiently induce TNF-α and IL-6 secretion, which may be due to the lower CPS levels than that of A1142. No obvious differences were found between the Ref-K57Δ*orf13*::A1142*orf13-tnp* and Ref-K57 strains (Fig. 5A,B). Notably, the complementation strain, A1142::Ref-K57*orf13,* induced TNF-α and IL-6 secretion more efficiently than the A1142 parent strain. Compared to the A1142 parental strain, A1142::Ref-K57*orf13* induced secretion of higher levels of TNF-α at 4 and 6 h post-infection (Fig. 5A). For IL-6 secretion, earlier induction by A1142::Ref-K57*orf13*, from <4 h post-infection, was observed (Fig. 5B). In addition, the levels of IL-6 induced by A1142::Ref-K57*orf13* at 6 h and 24 h post-infection were higher than those induced at respective time points by the A1142 parental strain. These results suggested that CPS acetylation enhanced K57 *K. pneumoniae* induction of pro-inflammatory cytokines.

To investigate underlying mechanisms, we determined the effect of CPS acetylation on the activation of NF-κB, JNK, p38 MAPK, and ERK1/2 in Raw 264.7 cell lysates by Western blotting (Fig. 5C). Consistent with the cytokine secretion data, the acetylation complementation strain induced the phosphorylation of NF-κB, JNK and p38 MAPK more efficiently than the A1142 parental strain. Therefore, acetylation of CPS possibly increased *K. pneumoniae*-induced cytokine production due to enhanced activation of JNK and MAPK signaling pathways.

### CPS acetylation decreases serum resistance of *K. pneumoniae* but promotes cell adhesion

**Figure 6:**
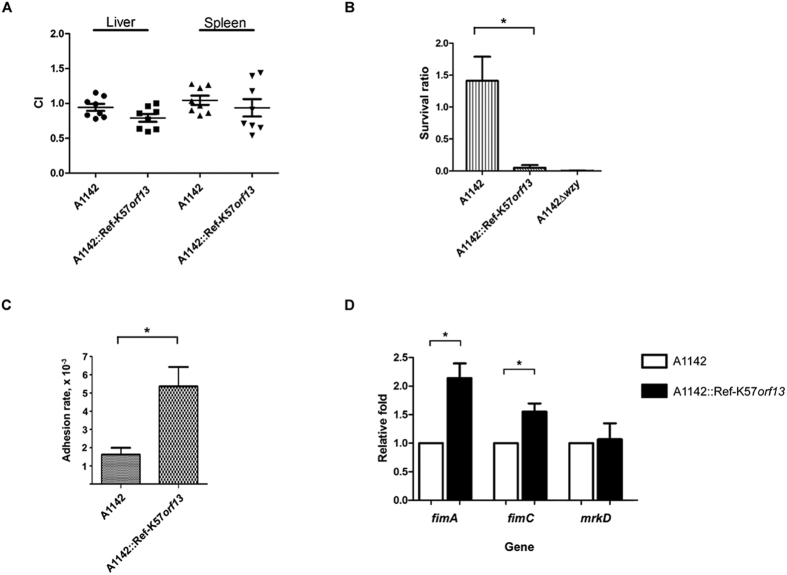
Effects of CPS acetylation on serum resistance and cell adhesion. (**A**) *In vivo* competition of the A1142 parent strain and the acetylation complementation strain (A1142::Ref-K57*orf13*) was tested in mice. Each test strain was compared with the A1142Δp*lacZ* mutant strain, and the ratio of LacZ-positive to LacZ-negative colonies in the liver or spleen of each mouse was determined. A1142Δ*placZ* was fully virulent as the parental A1142. The competitive index (CI) was defined as (outputΔp*lacZ*/output_test strain_)/(inputΔp*lacZ*/input_test strain_). Each symbol represents the CI for each inoculum, with the medians shown by bars. A1142 and A1142::Ref-K57*orf13, P* = 0.31 for liver and *P* = 0.81 for spleen, Wilcoxon signed rank test. (**B**) Serum resistance assays. *K. pneumoniae* strains were incubated with human serum (75%) at 37 °C for 1 h. The numbers of recovered CFU were determined, and the survival ratio was expressed as recovered CFU/inoculum CFU. The CPS-lacking mutant, A1142Δ*wzy*, which is sensitive to serum killing, was analyzed for comparison. Data are presented as mean ± SEM from three independent experiments. *P < 0.05, Student’s t test. (**C**) Adhesion of *K. pneumoniae* to Caco-2 cells. The adhesion rate was expressed as the proportion of the inoculum that adhered. Data are presented as mean ± SEM from three independent experiments. *P < 0.05, Student’s t test. (**D**) Relative expression of the fimbriae genes, *fimA, fimC*, and *mrkD*, was determined by RT-qPCR. The acetylation complementation strain, A1142::Ref-K57*orf13* (black bars, ◼), was compared to the A1142 parent strain (white bars, ◻, set as 1). Data are presented as mean ± SEM from three independent experiments. *P < 0.05, Student’s t test.

In mice inoculated with *K. pneumoniae* A1142 or the complementation strain, A1142::Ref-K57*orf13, in vivo* competition assays revealed no significant differences in the virulence between the strains (Fig. 6A; results of CFU viable counts shown in Figure S1). This assay reflected *in vivo* virulence of test strain in mice. Each test strain (LacZ-positive; blue colonies on the plates containing IPTG and X-Gal) was inoculated with the isogenic lacZ promoter deletion strain (white colonies) at a 1:1 ratio, and the numbers of bacterial colonies recovered from mice were compared. The colony numbers of the A1142 parent and A1142::Ref-K57*orf13* were both similar to that of A1142Δp*lacZ* (Figure S1), and no significant differences between A1142 and A1142::Ref-K57*orf13* in the competitive index (CI) were found (Fig. 6A). Thus, acetylation of CPS may not significantly change the *in vivo* virulence of A1142.

Given that the capsule protects *K. pneumoniae* from killing by the host sera in the immune system, we evaluated whether CPS acetylation affects the serum resistance of *K. pneumoniae. In vitro* serum resistance assays showed that although the A1142 strain grew in human serum (Fig. 6B), the A1142::Ref K57*orf13* strain became sensitive to serum-killing, indicating that CPS acetylation decreased the serum resistance of *K. pneumoniae* and diminished the protective properties of the K57 capsule. Acetyl modification of CPS may reduce the ability of *K. pneumoniae* to escape from the host immune system.

The capsule of *K. pneumoniae* has also been implicated in adhesion of bacteria to host cells^27^. We analyzed the effect of capsular acetylation on adhesion in a cell culture assay (Fig. 6C; results of CFU viable counts shown in Figure S2). Notably, A1142::Ref-K57*orf13* exhibited a higher rate of adhesion to human Caco-2 (intestinal epithelium-derived) cells than observed with the A1142 parental strain. Thus, CPS acetylation enhanced attachment of *K. pneumoniae* to host intestinal cells and may play a role in promoting its colonization and interactions with host cells.

Since India ink staining showed that the capsule thickness of A1142::Ref-K57*orf13* was not significantly changed, we investigated whether fimbriae, the surface factors important for *K. pneumoniae* adhesion, were altered. Expression of the type I fimbriae genes, *fimA* and *fimC,* and the type III fimbriae gene, *mrkD,* was determined by quantitative real-time reverse-transcription PCR (RT-qPCR) (Fig. 6D). Compared to the A1142 parental strain, the expression of *fimA* and *fimC* in A1142::Ref-K57*orf13* increased. No significant differences in *mrkD* were detected. Thus, the enhanced cell adhesion of the acetylation complementation strain could be due to higher levels of type I fimbriae.

## Discussion

Conventional definitions of the K serotypes of encapsulated bacteria assume that each type has a specific capsular polysaccharide structure and is a genetically distinct entity. The presence of variants in a K serotype has been described in some bacteria^28^,^29^,^30^. Here, we characterized a variant of the K57 capsular serotype, a type that is associated with *K. pneumoniae-*induced PLA. Our work revealed that this capsular variation reflected changes in CPS acetylation. Although acetylation has been widely studied in the capsule of several pathogenic bacteria, such as the *E. coli* K1 strain, *S. pneumoniae*, and *N. meningitidis*^20^,^21^,^22^,^31^,^32^, this modification has been poorly characterized in *Klebsiella* capsular types. Our studies identified a gene whose product is responsible for the acetylation of K57 CPS and demonstrated its impact. These results have implications for *K. pneumoniae* vaccine design and pathogenicity.

Based on PCR genotyping of the *cps* cluster and immunoserotyping, the PLA isolate, A1142, was defined as a K57 capsular type^14^. However, it harbored differences in the *cps* gene (*orf13*) encoding a putative acetyltransferase. In A1142, *orf13* is interrupted by insertion of a putative transposase-encoding gene and presumably inactivated. NMR, biochemical, and genetic analyses indicated that the intact *orf13* of Ref-K57 mediates CPS acetylation, and A1142 is a K57 variant that is deficient in CPS acetylation. Capsular variants involving acetylation have been described in other bacterial species. For instance, in *E. coli* with K1 capsular polysaccharides, variation in polysaccharide O-acetylation is mediated by a phase-variable gene encoding an O-acetyltransferase^32^,^33^. The determinant gene, *neuO*, is associated with the prophage CUS-3 and not part of the *cps* cluster. In *S. pneumoniae*, the polysaccharide structures of serotypes 11A and 11E appear to be identical except for the presence of O-acetylation^21^. This modification depends on the presence of the O-acetyltransferase-encoding gene, *wcjE,* in the 11A *cps* locus. Strains of serotype 11E contain various disruptive mutations in *wcjE* that result in inactivation of the gene. Another example is the reversible serotype switching between *S. pneumoniae* 15B and 15C, a variation that has been attributed to reversible slipped-strand mutations in the *wciZ* gene of the *cps* cluster. Because this gene encodes an O-acetyltransferase, strand slippage during replication results in phase-variable O-acetylation of capsule polysaccharides^34^,^35^. Our studies of *K. pneumoniae* demonstrated that varied acetylation of CPS in K57 strains was mediated by disruptive insertion of a putative transposase-encoding gene in the *cps* region at the site of the gene encoding acetyltransferase, indicating another mechanism of serotype variation in bacteria.

The capsular polysaccharides of *K. pneumoniae* are immunogenic. Antibodies targeting the capsule have been shown to provide hosts with increased resistance to capsulated pathogens^36^,^37^. Modifications of the CPS may influence the properties of polysaccharides. Acetylation affects the antigenicity or immunogenicity of other bacterial polysaccharides^32^,^38^,^39^,^40^, such as K1 *Escherichia coli*, groups W-135, Y, and C *meningococci*, and group B *Streptococcus* capsular polysaccharides. In agreement, our data demonstrated that the acetyl group influenced the immunoreactivity of *K. pneumoniae* K57 polysaccharides to anti-K57 antibodies. Immunoblotting of Ref-K57 CPS against anti-Ref-K57 antiserum showed that the immunoreactivity was significantly decreased by acetylation gene replacement (Ref-K57Δ*orf13*::A1142*orf13-tnp*). Therefore, the acetyl group may constitute part of the epitope of *K. pneumoniae* K57 CPS recognized by this antibody. CPS from A1142 displayed lower reactivity to anti-Ref-K57 antiserum, and complementation of acetylation enhanced the immunoreactivity of CPS in this strain. These results indicate that acetyl modification plays an important role in *K. pneumoniae* CPS antigenicity and antibody recognition, likely as the part of the polysaccharide epitope. Analysis of acetylation prevalence in *K. pneumoniae* clinical strains, including other PLA predominant capsular types, merits further survey. These results also suggest that polysaccharide modifications should be considered in the development of capsule-based vaccines for *K. pneumoniae*.

We also demonstrated the effects of CPS acetylation on innate immune responses. Notably, acetylation enhanced K57 *K. pneumoniae* induction of TNF-α and IL-6. This result is consistent with the previous demonstration that chemical removal of O-acetyl and O-pyruvyl groups from extracted K1 CPS attenuates the induction of pro-inflammatory cytokines^23^. In the same study, K1 CPS was shown to induce cytokine expression through activation of Toll-like receptor 4 (TLR4) and MAPK signaling pathways^23^. Our studies of K57 *K. pneumoniae* further revealed a role for CPS acetylation in host signal transduction. Thus, acetylation may improve CPS induction of cytokines via activating cell signaling pathways, including JNK and MAPK. We propose that acetylation possibly mediates the interactions between polysaccharides and host receptors to initiate cell signaling pathways, therefore, contributing to polysaccharide induction of cytokine expression and innate immune responses in the host.

Alternation of surface polysaccharide architecture by acetylation has been proposed to influence the virulence of several bacterial pathogens, including *E. coli, N. meningitidis*, and *Staphylococcus aureus*^21^,^22^,^31^. Epidemiological association of O-acetylated CPS and increased virulence was also reported in bacteremia-inducing *E. coli* K1 strains^21^. One study of *S. aureus* serotype 5 showed that a mutant lacking O-acetylation has reduced bacterial virulence in opsonophagocytic assays and in mice^31^. Our study of *K. pneumoniae* suggested that capsular acetylation contributed to pathogenicity; however, complementation of acetylation did not significantly change the *in vivo* virulence of the non-acetylated PLA strain A1142 in mice. Instead, acetylation influenced *K. pneumoniae* resistance against human serum and adhesion to human intestinal cells although with opposing effects. While acetylation reduced serum resistance of A1142, it promoted bacterial adhesion to the host cells, which may explain the unchanged *in vivo* virulence observed in the mouse experiments. Additionally, many other factors could also be involved in the *in vivo* results, i.e. an overall consequence. For example, *K. pneumoniae* might regulate their CPS production or acetylation levels in the host environment, or acetylation could affect other unidentified host responses. Our data imply that acetylation in *K. pneumoniae* K57 has different impacts on bacterial survival and adaptation. This capsular modification may play a variety of roles in *K. pneumoniae* pathogenicity, requiring further studies using different clinical strains and other K-type *K. pneumoniae*.

We demonstrated that acetylation of CPS promoted cell adhesion of *K. pneumoniae*. No significant changes in CPS production and the capsule thickness were found. Thus, one possibility for the better cell adhesion could be that acetylation enhanced interactions between bacterial surfaces with the host cells despite expressing similar amounts of CPS. RT-qPCR revealed that the expression of type I fimbriae genes was higher in the acetylated strain. Therefore, one explanation for increased adhesion might be due to more production of fimbriae in the acetylated CPS strain. Why and how complementation of acetylation could affect fimbriae expression is still unclear. Potential gene regulation networks or interplay between *cps* and *fim* genes should be further characterized.

Capsular typing of *K. pneumoniae* is important for determining its prevalence for epidemiological research. The inability to determine the types for some clinical isolates is not unusual. For example, an Australian survey using antisera for typing reported that out of 293 *K. pneumoniae* isolates, 88 (30%) could not be typed, and 54 (18%) had a positive reaction for more than one capsular type^41^. *Klebsiella* capsules can be typed using different methods, and each has some limitations that can preclude serotype determination in clinical isolates^42^. The presence of CPS variants like that described in the present study may provide another explanation. Serological diagnosis is commonly used to determine *Klebsiella* capsular serotypes of clinical isolates often exhibit different sensitivities. Varied acetylation of CPS may render some clinical strains less immunoreactive to antiserum. Since modifications increase the complexity of the capsule, investigators may need to employ more than one approach to determine the type accurately.

In summary, DNA sequence analysis of the *cps* cluster permitted identification of a candidate CPS acetyltransferase-encoding gene with distinct alleles in different K57 strains. We correlated this genetic variation with acetylation-mediated changes in *K. pneumoniae* PLA-related capsular serotype K57. We further showed the impacts of acetylation on *K. pneumoniae* CPS immunoreactivity, host innate response, serum resistance, and host cell adhesion. We suggest that acetylation contributes to the diversity and antigenic heterogeneity of the capsule and *K. pneumoniae*-host interactions. Our findings increase our knowledge about the variety of *Klebsiella* CPS and provide further insights into the role of capsular modification in bacterial pathogenesis.

## Methods

### Bacterial strains, *cps*-PCR, and *cps* sequencing

The Ref-K57 strain, 4425/51, was purchased from the Statens Serum Institute (Copenhagen, Denmark). Twenty-two *K. pneumoniae* K57 clinical isolates, including PLA and bacteremia strains, were collected from the National Taiwan University Hospital, En Chu Kong Hospital, Chang Gung Memorial Hospital, and Taipei Veterans General Hospital in Taiwan^14^,^19^,^43^,^44^, and were gifts from Hong Kong and Finland^14^,^44^. The K57 capsular type of the clinical strains was determined using *wzy*-targeting, PCR-based *cps* genotyping^14^,^43^. PCR analysis of *orf13*, the *cps* gene encoding a putative acetyltransferase, was performed using the following primers: K57-*orf13*-F (atgggtaaaaatatcaaagagcg) and K57-*orf13*-R (ttagaataggaaccataatcttttcc).

For *cps* sequencing, the *cps* region of the Ref-K57 strain (between the conserved genes, *galF* and *gnd*) was amplified as previously described^14^. The products were sequenced by primer walking, providing DNA sequences for the *cps* regions. Genes were annotated by NCBI-BLAST.

### Analysis of CPS acetylation

The degree of acetylation was quantified according to a modified Hestrin method^24^,^25^. Extracellular polysaccharides were extracted with hot phenol as previously reported^19^ and further purified by ethanol re-precipitation. Each sample was resuspended in water (0.5 mL) and combined with 1 mL of alkaline hydroxylamine (1M hydroxylamine in 1.75M NaOH), and the mixture was incubated for 30 min before addition of 0.5 mL each of 4M HCl and 0.37M FeCl_3_ in 0.1M HCl. Absorbance was measured at 504 nm (*A*_504_). For comparison, data are presented as A_504_ normalized to 50 μg of CPS for each sample.

To analyze potential acetylation of *K. pneumoniae* CPS using ^1^H NMR^23^, the O-antigen-lacking mutants, Ref-K57Δ*wecA* and A1142Δ*wecA,* were constructed using an unmarked deletion method with the temperature-sensitive plasmid pKO3-Km^45^ to avoid the effects of sugars from lipopolysaccharide (LPS). *K. pneumoniae* CPS was isolated according to the previously described methods^23^ with modifications as needed. Briefly, bacteria suspended in distilled water were heated for 10 min at 100 °C to release capsular materials; CPS was precipitated with 80% (v/v) acetone at 4 °C. Dried precipitate was resuspended in 20mM Tris-HCl, pH 7.5, and treated with ribonuclease, deoxyribonuclease I, and proteinase K. The sample was then dialyzed extensively against water using an 80-kDa cutoff membrane and lyophilized. The CPS was further purified on a TSK HW-65F column, followed by dialysis and re-lyophilization. Sugar composition was determined by methanolysis and trimethylsilylation, followed by GC-MS analysis^46^.

### Quantification of CPS

K57 CPS, which contains uronic acid, was quantified according to previously described methods^47^,^48^. Briefly, extracted samples from the equivalent amounts of bacterial overnight cultures were resuspended in 0.1 mL of water and combined with 1.2 mL of 12.5 mM tetraborate in concentrated H_2_SO_4_. After vigorous vortexing, the mixture was boiled for 5 min. After cooling, 20 μL of 0.15% 3-hydroxydiphenol (Sigma-Aldrich, St. Louis, MO) was added. Then, the absorbance at 520 nm was measured.

### Immunoblotting of CPS

For immunoblot analysis of CPS, rabbit anti-K57 antiserum was purchased from the Statens Serum Institute. Extracted polysaccharide samples were vacuum-spotted onto a nitrocellulose membrane using a slot blot device. The membrane was overlapped with a piece of filter, and both were rinsed with Western transfer buffer containing 47.8 mM Tris, 38.6 mM glycine, 20% MeOH, and 0.037% sodium dodecyl sulfate. The membrane was dried, and non-specific sites were blocked by soaking the membrane in 1× phosphate-buffered saline with 0.5% Tween 20 (PBST) plus 5% milk for 1 h at room temperature. The membrane then was incubated with anti-K57 antiserum (1:1000 dilution) dissolved in PBST plus milk at 4 °C overnight, washed four times with PBST for 10 min each, incubated with the secondary antibody conjugated with horseradish peroxidase (goat anti-rabbit IgG-HRP, 1:10 000) for 1 h at room temperature, and washed three times with PBST for 10 min each. The ECL reagent was added for 3 min, and the membrane was exposed to X-ray film in the dark. To quantify the immunoreactivity, the resulting bands were analyzed by densitometry using Image J software (NIH, USA).

### Gene replacement and complementation in *K. pneumoniae*

The isogenic replacement mutant of Ref-K57 was constructed using the temperature-sensitive plasmid, pKO3-Km^43^,^45^. In this replacement mutant, the coding region of the putative acetyltransferase (*orf13*) in the *cps* locus of Ref-K57 was replaced by the corresponding gene (disrupted by an ORF encoding a putative transposase) from A1142 (residues 15933–17975). For chromosomal complementation of *orf13* in the *cps* cluster of A1142, a single copy of the Ref-K57 *orf13* was cloned into the intergenic region between *wbaZ* and *gnd* using the pKO3-Km vector according to a previously reported method^49^. The gene replacement or complementation was confirmed by PCR analysis and DNA sequencing.

### Analysis of pro-inflammatory cytokine secretion and cell signaling

RAW 264.7 murine macrophage-like cells were grown in Dulbecco’s modified Eagle’s medium (DMEM) supplemented with 10% fetal bovine serum (FBS) and 1× penicillin/streptomycin (PAA) and maintained at 37 °C in a humidified incubator at 95% air-5% CO_2_. Culture medium and supplements were obtained from GIBCO/BRL (Gaithersburg, MD, USA). For infection, cells (1 × 10^6^ cells per well) in FBS-free DMEM medium in 24-well plates were infected with mid-log-phase (*A*_600_ = 0.4 to 0.6) *K. pneumoniae* at a multiplicity of infection of 10 (MOI 10; bacteria/cells). After 1 h of infection, the cells were treated with 100 μg/mL of gentamicin and supernatants were collected at various time points as indicated. The levels of mouse TNF-α and IL-6 in culture supernatants were measured by enzyme-linked immunosorbent assay (ELISA) kits (R&D Systems, Minneapolis, MN, USA) according to the manufacturer’s instructions.

For Western blot detection of cell signaling, RAW 264.7 cells were harvested after *K. pneumoniae* induction (as described above). Total cellular protein extracts were obtained by lysing cells in ice cold RIPA buffer, followed by centrifugation and protein quantification using a Coomassie blue protein assay kit (Bio-Rad, Hercules, CA, USA). Equal amounts of the protein extracts were subjected to SDS-PAGE electrophoresis and subsequent electrotransfer onto a PVDF membrane. After blocking with 5% skimmed milk in PBST for 1 h at room temperature, the blots were incubated with primary antibodies overnight at 4 °C. Antibodies against phospho-ERK1/2 (Thr202/185 and Tyr204/187), phospho-p38 MAPK (Thr180/Tyr182), phospho-JNK (Thr183/Tyr185), phospho-NF-κB p65 (Ser536) and those against respective total proteins were obtained from Cell Signaling (Beverly, MA, USA). Antibodies against β-actin were from Santa Cruz Biotechnology (Santa Cruz, CA, USA). After washes with PBST, the blots were incubated with HRP-conjugated secondary antibodies (Jackson Immuno Research Laboratories, West Grove, PA, USA), and the signals were detected using a digital imaging system (UVP, Upland, CA, USA).

### *In vivo* competition assays

*In vivo* competition of *K. pneumoniae* in mice was analyzed as outlined in previous work^49^. Briefly, the parental A1142 or A1142::Ref-K57*orf13* strains were mixed with the isogenic *lacZ* promoter deletion mutant (A1142Δp*lacZ*) at a 1:1 ratio, and a per-mouse dose of 5 × 10^6^ colony-forming units (CFUs) in 100 μL of saline solution was inoculated intraperitoneally into 5-week-old BALB/c mice. Each mouse was killed 24 h post-inoculation, and the liver and spleen were removed and homogenized in 1× PBS. The number of LacZ-positive and LacZ-negative colonies on LB plates containing 1 mM IPTG/mL and 50 μg/mL of X-Gal were counted. The competitive index (CI) was defined as (outputΔp*lacZ*/output_test strain_)/(inputΔp*lacZ*/input_test strain_). All animal experiments were carried out in accordance with the Guide for the Care and Use of Laboratory Animals, Institute of Laboratory Animal Resources Commission on Life Sciences National Research Council, USA, and all animal protocols were approved by the Institutional Animal Care and Use Committee (IACUC) of the National Taiwan University College of Medicine (NTUCM).

### Serum resistance assays

The serum resistance of the *K. pneumoniae* strains was analyzed as previously described^44^. Briefly, an inoculum of 2.5 × 10^4^ CFU bacteria in 25 μL was mixed with 75 μL human serum from healthy volunteers. The mixture was incubated at 37 °C for 1 h. After serial dilution and plating, the numbers of CFU were determined. The survival ratio was calculated; values of ≧1 were defined as serum resistance. The experiments were repeated independently three times.

### Cell adhesion assays

Adhesion assays using human enterocyte-like Caco-2 cells were performed according to previously described methods^50^. Caco-2 cells were maintained in DMEM medium supplemented with 10% heat-inactivated FBS and 1% nonessential amino acids. For adhesion assays, the cells seeded in 24-well plates (~5 × 10^5^ cells per well) and were prewashed with Hanks’ Balanced Salt Solution (HBSS). Mid-log-phase (*A*_600_ = 0.4 to 0.6) *K. pneumoniae* in FBS-free DMEM medium were added to each well at a MOI of 50. After a 20-min incubation in a humidified 5% CO_2_ atmosphere at 37 °C, the wells were washed with HBSS three times, and bacteria were released by the addition of 0.2% Triton X-100 (Sigma-Aldrich). The experiments were conducted in duplicate and repeated independently three times.

### Quantitative real-time reverse-transcription PCR (RT-qPCR)

*K. pneumoniae* gene expression was determined by RT-qPCR^49^. Total RNA from *K. pneumoniae* strains was isolated using the RNeasy Mini kit (Qiagen, Valencia, CA, USA) with DNase I to remove contaminating genomic DNA. A 400-ng sample of purified total RNA was reverse-transcribed and amplified by PCR with SYBR green dye (Invitrogen) in an ABI 7900 thermocycler (Applied Biosystems, Foster City, CA, USA). For each gene, the calculated threshold cycle (Ct) was normalized to the Ct of the 23S ribosomal RNA gene from the same complementary DNA sample. The relative RNA expression was calculated based on the ΔΔCt value. Primers used are listed in Table S1.

### Statistical analysis

Comparisons of mean values were assessed by a two-tailed Student’s t test using Prism 5 (Graphpad) software. *P* values of <0.05 were considered significant.

## Additional Information

**How to cite this article**: Hsu, C.-R. *et al*. Identification of a capsular variant and characterization of capsular acetylation in *Klebsiella pneumoniae* PLA-associated type K57. *Sci. Rep.* **6**, 31946; doi: 10.1038/srep31946 (2016).

## Supplementary Material

Supplementary Information

## Figures and Tables

**Table 1:** Gene annotation of the *cps* region in Ref-K57 and comparison with that in A1142.

Ref-K57			Predicted functions^a^	A1142^b^			DNA sequence similarity^c^
Name	Location (nt)^d^	Product size (aa)^e^		Name	Location (nt)	Product size (aa)	
*galF*	1–903	300	UDP-glucose pyrophosphorylase	*galF*	1–891	296	93.2% (842/903)
*orf2*	1297–1926	209	Acid phosphatase homologue	*orf2*	1283–1912	209	91.7% (578/630)
*wzi*	2805–4325	506	Surface assembly of capsule	*wzi*	2872–4311	479	1299/1521 (85.4%)
*wza*	4471–5604	377	Putative capsule polysaccharide export protein	*wza*	4457–5590	377	1067/1137 (93.8%)
*wzb*	5601–6044	147	Protein tyrosine phosphatase	*wzb*	5596–6030	144	435/444 (98.0%)
*wzc*	6061–8223	720	Tyrosine-protein kinase	*wzc*	6047–8209	720	2149/2163 (99.4%)
*wbaP*	8296–9726	476	Undecaprenolphosphate hexose-1-P transferase	*wbaP*	8282–9712	476	1423/1431 (99.4%)
*wzx*	9750–11213	487	Flippase	*wzx*	9736–11199	487	1458/1464 (99.6%)
*orf9*	11551–12336	261	Glycosyltransferase-like	*orf9*	11537–12322	261	772/786 (98.2%)
*orf10*	12345–13544	399	Glycosyl transferase	*orf10*	12331–13425	364	1085/1200 (90.4%)
*wzy*	13561–14487	308	O antigen and lipid-linked capsular repeat unit polymerase	*wzy*	13440–14618	392	908/1176 (77.2%)
*orf12*	14617–15927	436	Gluconolactonase	*orf12*	14650–15912	420	1254/1311 (95.7%)
*orf13*	15948–16928	326	Acyltransferase family protein	*orf13-5*′	15933–16233	96	255/288 (88.5%);
			Transposase	*orf14*	16519–17193	224	
			Acyltransferase family protein	*orf13-3*′	17367–17978	203	600/612 (98.0%)
*wbaZ*	17024–18163	379	Mannosyltransferase	*wbaZ*	18092–19243	383	1110/1170 (94.9%)
*gnd*	18339–19745	468	Gluconate-6-phosphate dehydrogenase	*gnd*	19389–20795	468	1383/1407 (98.3%)

^a^Determined by NCBI BLASTP.

^b^Published A1142 *cps* sequences from Pan *et al*.^14^.

^c^Compared using the sequence alignment program, ClustalW2 (http://www.ebi.ac.uk/Tools/msa/clustalw2/).

^d^nt, nucleotide. The first nucleotide of *galF* is defined as position 1.

^e^aa, amino acid.

## References

[b1] Ko W. C. . Community-acquired Klebsiella pneumoniae bacteremia: global differences in clinical patterns. Emerg Infect Dis 8, 160–166 (2002). 11897067 10.3201/eid0802.010025 PMC2732457

[b2] Lin Y. T., Jeng Y. Y., Chen T. L. & Fung C. P. Bacteremic community-acquired pneumonia due to Klebsiella pneumoniae: clinical and microbiological characteristics in Taiwan, 2001–2008. BMC Infect Dis 10, 307 (2010). 20973971 10.1186/1471-2334-10-307 PMC2987304

[b3] Podschun R. & Ullmann U. Klebsiella spp. as nosocomial pathogens: epidemiology, taxonomy, typing methods, and pathogenicity factors. Clin Microbiol Rev 11, 589–603 (1998). 9767057 10.1128/cmr.11.4.589 PMC88898

[b4] Tsai F. C., Huang Y. T., Chang L. Y. & Wang J. T. Pyogenic liver abscess as endemic disease, Taiwan. Emerg Infect Dis 14, 1592–1600 (2008). 18826824 10.3201/eid1410.071254 PMC2609891

[b5] Chung D. R. . Emerging invasive liver abscess caused by K1 serotype Klebsiella pneumoniae in Korea. J Infect 54, 578–583 (2007). 17175028 10.1016/j.jinf.2006.11.008

[b6] Lederman E. R. & Crum N. F. Pyogenic liver abscess with a focus on Klebsiella pneumoniae as a primary pathogen: an emerging disease with unique clinical characteristics. Am J Gastroenterol 100, 322–331 (2005). 15667489 10.1111/j.1572-0241.2005.40310.x

[b7] Yang C. C., Yen C. H., Ho M. W. & Wang J. H. Comparison of pyogenic liver abscess caused by non-Klebsiella pneumoniae and Klebsiella pneumoniae. J Microbiol Immunol Infect 37, 176–184 (2004). 15221038

[b8] Fung C. P. . A global emerging disease of Klebsiella pneumoniae liver abscess: is serotype K1 an important factor for complicated endophthalmitis? Gut 50, 420–424 (2002). 11839725 10.1136/gut.50.3.420 PMC1773126

[b9] Chuang H. C. . Clinical and bacteriological characteristics of pyogenic liver abscess in non-diabetic patients. J Microbiol Immunol Infect 42, 385–392 (2009). 20182667

[b10] Thomsen R. W., Jepsen P. & Sorensen H. T. Diabetes mellitus and pyogenic liver abscess: risk and prognosis. Clin Infect Dis 44, 1194–1201 (2007). 17407038 10.1086/513201

[b11] Cortes G. . Molecular analysis of the contribution of the capsular polysaccharide and the lipopolysaccharide O side chain to the virulence of Klebsiella pneumoniae in a murine model of pneumonia. Infect Immun 70, 2583–2590 (2002). 11953399 10.1128/IAI.70.5.2583-2590.2002 PMC127904

[b12] Mizuta K. . Virulence for mice of Klebsiella strains belonging to the O1 group: relationship to their capsular (K) types. Infect Immun 40, 56–61 (1983). 6187694 10.1128/iai.40.1.56-61.1983 PMC264817

[b13] Fang C. T. . Klebsiella pneumoniae genotype K1: an emerging pathogen that causes septic ocular or central nervous system complications from pyogenic liver abscess. Clin Infect Dis 45, 284–293 (2007). 17599305 10.1086/519262

[b14] Pan Y. J. . Capsular polysaccharide synthesis regions in Klebsiella pneumoniae serotype K57 and a new capsular serotype. J Clin Microbiol 46, 2231–2240 (2008). 18508935 10.1128/JCM.01716-07 PMC2446917

[b15] Struve C., Bojer M., Nielsen E. M., Hansen D. S. & Krogfelt K. A. Investigation of the putative virulence gene magA in a worldwide collection of 495 Klebsiella isolates: magA is restricted to the gene cluster of Klebsiella pneumoniae capsule serotype K1. J Med Microbiol 54, 1111–1113 (2005). 16192445 10.1099/jmm.0.46165-0

[b16] Yu W. L. . Comparison of prevalence of virulence factors for Klebsiella pneumoniae liver abscesses between isolates with capsular K1/K2 and non-K1/K2 serotypes. Diagn Microbiol Infect Dis 62, 1–6 (2008). 18486404 10.1016/j.diagmicrobio.2008.04.007

[b17] Erbing C., Kenne L., Lindberg B. & Lonngren J. Structural studies of the capsular polysaccharide from Klebsiella Type 1. Carbohydr Res 50, 115–120 (1976). 975115 10.1016/s0008-6215(00)84088-4

[b18] Ayling-Smith B. & Pitt T. L. State of the art in typing: Klebsiella spp. J Hosp Infect 16, 287–295 (1990). 1980501 10.1016/0195-6701(90)90001-5

[b19] Chuang Y. P., Fang C. T., Lai S. Y., Chang S. C. & Wang J. T. Genetic determinants of capsular serotype K1 of Klebsiella pneumoniae causing primary pyogenic liver abscess. J Infect Dis 193, 645–654 (2006). 16453259 10.1086/499968

[b20] Calix J. J. & Nahm M. H. A new pneumococcal serotype, 11E, has a variably inactivated wcjE gene. J Infect Dis 202, 29–38 (2010). 20507232 10.1086/653123 PMC2880655

[b21] Frasa H. . Escherichia coli in bacteremia: O-acetylated K1 strains appear to be more virulent than non-O-acetylated K1 strains. J Clin Microbiol 31, 3174–3178 (1993). 7508454 10.1128/jcm.31.12.3174-3178.1993 PMC266371

[b22] Lee H. J. . Structural and kinetic characterizations of the polysialic acid O-acetyltransferase OatWY from Neisseria meningitidis. J Biol Chem 284, 24501–24511 (2009). 19525232 10.1074/jbc.M109.006049 PMC2782042

[b23] Yang F. L. . Structure and immunological characterization of the capsular polysaccharide of a pyrogenic liver abscess caused by Klebsiella pneumoniae: activation of macrophages through Toll-like receptor 4. J Biol Chem 286, 21041–21051 (2011). 21478151 10.1074/jbc.M111.222091 PMC3122165

[b24] Hestrin S. The reaction of acetylcholine and other carboxylic acid derivatives with hydroxylamine, and its analytical application. J Biol Chem 180, 249–261 (1949). 18133390

[b25] Naran R., Chen G. & Carpita N. C. Novel rhamnogalacturonan I and arabinoxylan polysaccharides of flax seed mucilage. Plant Physiol 148, 132–141 (2008). 18667723 10.1104/pp.108.123513 PMC2528086

[b26] Kamerling J. P., Lindberg B., Lonngren J. & Nimmich W. Structural studies of the Klebsiella type 57 capsular polysaccharide. Acta Chem Scand B 29, 593–598 (1975). 1166725 10.3891/acta.chem.scand.29b-0593

[b27] Sahly H. . Capsule impedes adhesion to and invasion of epithelial cells by Klebsiella pneumoniae. Infect Immun 68, 6744–6749 (2000). 11083790 10.1128/iai.68.12.6744-6749.2000 PMC97775

[b28] Orskov F. . Form variation in Escherichia coli K1: determined by O-acetylation of the capsular polysaccharide. J Exp Med 149, 669–685 (1979). 372481 10.1084/jem.149.3.669 PMC2184817

[b29] Lewis A. L., Nizet V. & Varki A. Discovery and characterization of sialic acid O-acetylation in group B Streptococcus. Proc Natl Acad Sci USA 101, 11123–11128 (2004). 15263085 10.1073/pnas.0403010101 PMC503750

[b30] Zartler E. R. . Structure of the capsular polysaccharide of pneumococcal serotype 11A reveals a novel acetylglycerol that is the structural basis for 11A subtypes. J Biol Chem 284, 7318–7329 (2009). 19114709 10.1074/jbc.M807952200 PMC2652282

[b31] Bhasin N. . Identification of a gene essential for O-acetylation of the Staphylococcus aureus type 5 capsular polysaccharide. Mol Microbiol 27, 9–21 (1998). 9466251 10.1046/j.1365-2958.1998.00646.x

[b32] Deszo E. L., Steenbergen S. M., Freedberg D. I. & Vimr E. R. Escherichia coli K1 polysialic acid O-acetyltransferase gene, neuO, and the mechanism of capsule form variation involving a mobile contingency locus. Proc Natl Acad Sci USA 102, 5564–5569 (2005). 15809431 10.1073/pnas.0407428102 PMC555961

[b33] Stummeyer K. . Evolution of bacteriophages infecting encapsulated bacteria: lessons from Escherichia coli K1-specific phages. Mol Microbiol 60, 1123–1135 (2006). 16689790 10.1111/j.1365-2958.2006.05173.x

[b34] Jones C. & Lemercinier X. Full NMR assignment and revised structure for the capsular polysaccharide from Streptococcus pneumoniae type 15B. Carbohydr Res 340, 403–409 (2005). 15680595 10.1016/j.carres.2004.12.009

[b35] van Selm S., van Cann L. M., Kolkman M. A., van der Zeijst B. A. & van Putten J. P. Genetic basis for the structural difference between Streptococcus pneumoniae serotype 15B and 15C capsular polysaccharides. Infect Immun 71, 6192–6198 (2003). 14573636 10.1128/IAI.71.11.6192-6198.2003 PMC219561

[b36] Meng C. . Development of 5-valent conjugate pneumococcal protein A-Capsular polysaccharide pneumococcal vaccine against invasive pneumococcal disease. Microb Pathog 47, 151–156 (2009). 19467319 10.1016/j.micpath.2009.05.004

[b37] Robbins J. B. . Prevention of invasive bacterial diseases by immunization with polysaccharide-protein conjugates. Curr Top Microbiol Immunol 146, 169–180 (1989). 2659266 10.1007/978-3-642-74529-4_18

[b38] Fattom A. I., Sarwar J., Basham L., Ennifar S. & Naso R. Antigenic determinants of Staphylococcus aureus type 5 and type 8 capsular polysaccharide vaccines. Infect Immun 66, 4588–4592 (1998). 9746554 10.1128/iai.66.10.4588-4592.1998 PMC108565

[b39] Fusco P. C., Farley E. K., Huang C. H., Moore S. & Michon F. Protective meningococcal capsular polysaccharide epitopes and the role of O acetylation. Clin Vaccine Immunol 14, 577–584 (2007). 17376859 10.1128/CVI.00009-07 PMC1865638

[b40] Arakere G. & Frasch C. E. Specificity of antibodies to O-acetyl-positive and O-acetyl-negative group C meningococcal polysaccharides in sera from vaccinees and carriers. Infect Immun 59, 4349–4356 (1991). 1937795 10.1128/iai.59.12.4349-4356.1991 PMC259048

[b41] Jenney A. W. . Seroepidemiology of Klebsiella pneumoniae in an Australian Tertiary Hospital and its implications for vaccine development. J Clin Microbiol 44, 102–107 (2006). 16390956 10.1128/JCM.44.1.102-107.2006 PMC1351949

[b42] Hsu C. R., Lin T. L., Pan Y. J., Hsieh P. F. & Wang J. T. Isolation of a bacteriophage specific for a new capsular type of Klebsiella pneumoniae and characterization of its polysaccharide depolymerase. PloS One 8, e70092 (2013). 23936379 10.1371/journal.pone.0070092 PMC3732264

[b43] Hsu C. R., Lin T. L., Chen Y. C., Chou H. C. & Wang J. T. The role of Klebsiella pneumoniae rmpA in capsular polysaccharide synthesis and virulence revisited. Microbiology(SGM) 157, 3446–3457 (2011). 10.1099/mic.0.050336-0 21964731

[b44] Fang C. T., Chuang Y. P., Shun C. T., Chang S. C. & Wang J. T. A novel virulence gene in Klebsiella pneumoniae strains causing primary liver abscess and septic metastatic complications. J Exp Med 199, 697–705 (2004). 14993253 10.1084/jem.20030857 PMC2213305

[b45] Hsieh P. F., Lin T. L., Lee C. Z., Tsai S. F. & Wang J. T. Serum-induced iron-acquisition systems and TonB contribute to virulence in Klebsiella pneumoniae causing primary pyogenic liver abscess. J Infect Dis 197, 1717–1727 (2008). 18433330 10.1086/588383

[b46] Yang F. L. . Structural determination of the polar glycoglycerolipids from thermophilic bacteria Meiothermus taiwanensis. Eur J Biochem/FEBS 271, 4545–4551 (2004). 10.1111/j.1432-1033.2004.04415.x 15560795

[b47] Blumenkrantz N. & Asboe-Hansen G. New method for quantitative determination of uronic acids. Anal Biochem 54, 484–489 (1973). 4269305 10.1016/0003-2697(73)90377-1

[b48] Domenico P., Schwartz S. & Cunha B. A. Reduction of capsular polysaccharide production in Klebsiella pneumoniae by sodium salicylate. Infect Immun 57, 3778–3782 (1989). 2680983 10.1128/iai.57.12.3778-3782.1989 PMC259904

[b49] Hsieh P. F., Lin H. H., Lin T. L. & Wang J. T. CadC regulates cad and tdc operons in response to gastrointestinal stresses and enhances intestinal colonization of Klebsiella pneumoniae. J Infect Dis 202, 52–64 (2010). 20497056 10.1086/653079

[b50] Hsu C. R. . Klebsiella pneumoniae translocates across the intestinal epithelium via Rho GTPase- and phosphatidylinositol 3-kinase/Akt-dependent cell invasion. Infect Immun 83, 769–779 (2015). 25452552 10.1128/IAI.02345-14 PMC4294243

