# Supplementary material for: Identification of a capsular variant and characterization of capsular acetylation in Klebsiella pneumoniae PLA-associated type K57
Source: Sci Rep. 2016 Aug 23;6:31946. doi: 10.1038/srep31946 (PMC4994043; doi:10.1038/srep31946)
Supplement: Supplementary Information [file srep31946-s1.pdf]

Supplementary information

SREP-16-10568A

Title: Identification of a capsular variant and characterization of capsular acetylation in Klebsiella pneumoniae PLA-associated type K57

Authors: Chun-Ru Hsu, Chun-Hsing Liao, Tzu-Lung Lin, Han-Ru Yang, Feng-Ling Yang, Pei-Fang Hsieh, Shih-Hsiung Wu, Jin-Town Wang

Figure S1

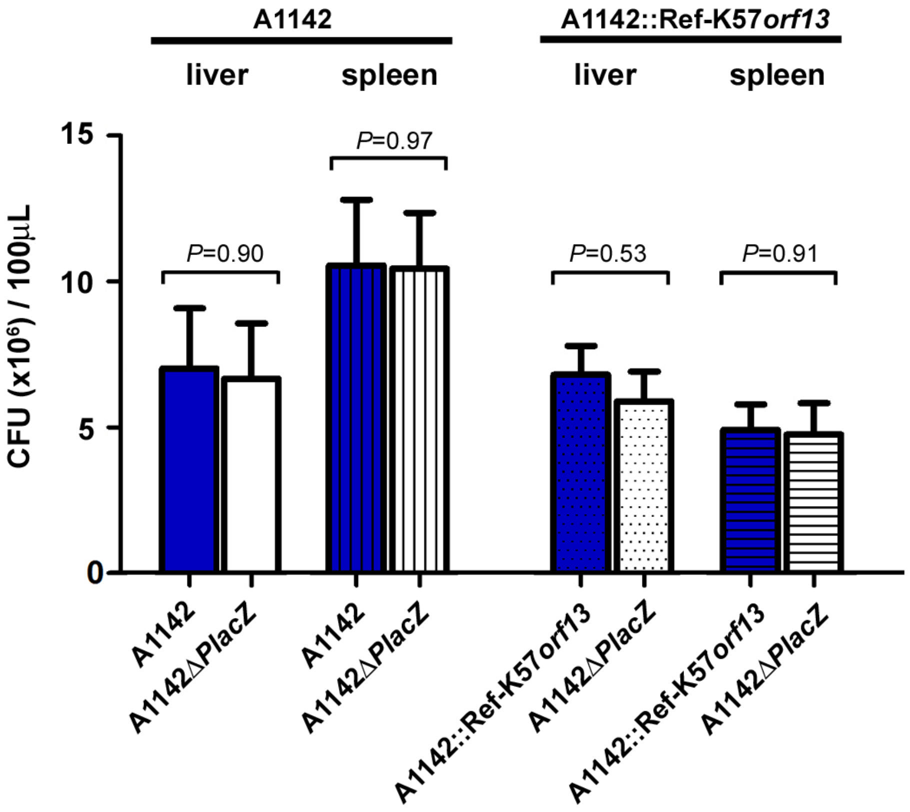

Figure S1. CFU counts in the *in vivo* competition assays.

In vivo virulence of the A1142 parent strain and acetylation complementation strain (A1142::Ref-K57*orf13*) was tested in mice. Each LacZ-positive test strain was mixed with the isogenic lacZ promoter deletion mutant (A1142ΔplacZ) at a 1:1 ratio and inoculated into mice. Bacterial colonies in the contents of the liver or spleen of each mouse were recovered 24 h post-inoculation on LB plates containing IPTG and X-Gal. The number of LacZ-positive (blue) to LacZ-negative (white) colonies was counted and compared. Data are mean ± SEM from eight mice.

Figure S2

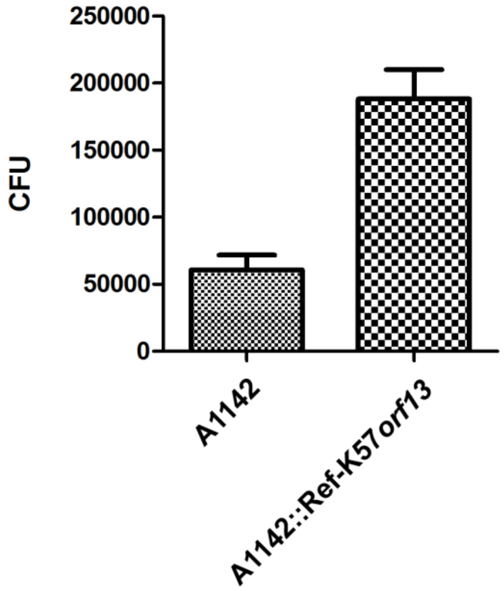

Figure S2. CFU counts in the adhesion assays.

Mid-log-phase *K. pneumoniae* (as indicated) was incubated with 5×10^5 Caco-2 cells at a MOI of 50 (inoculum) for 15 min, followed by a HBSS wash, Triton X-100 lysis and plating cell-adherent bacteria on LB agar. The results are expressed in the total recovered cell-adherent CFUs without normalization with the inoculum CFUs. Data are presented as mean ± SEM from three independent experiments. For A1142 and A1142::Ref-K57orf13, $P<0.05$, Student's t test.

Table S1. Specific primers used for RT-qPCR analysis.

Name	Sequence (5´-3´)	Purpose in this study	References or source
fimA-218F	TCGATCAAACCGTTCAGTTAGG	Real-time PCR for *fimA*	This study
fimA-330R	ACCGTAGTGTCGCAATCATC	Real-time PCR for *fimA*	This study
fimC-21F	TGTGAGAAAAACGGCAACGA	Real-time PCR for *fimC*	49
fimC-83R	CTGGCGGCGAAAAGCA	Real-time PCR for *fimC*	49
mrkD-344F	TCGTCTATCCCGACGTCTTT	Real-time PCR for *mrkD*	This study
mrkD-493R	CCAGTCGTAGGAGGTGTACTTA	Real-time PCR for *mrkD*	This study
KP-23-F	GGTTAAGCGACTAAGCGTACACGGT	Real-time PCR for 23S rRNA	49
KP-23-R	ACGAGGCGCTACCTAAATAGCTTTC	Real-time PCR for 23S rRNA	49
